# Lycopene ameliorates diabetes-induced pancreatic, hepatic, and renal damage by modulating the JAK/STAT/SOCS signaling pathway in rats

**DOI:** 10.22038/ijbms.2025.79979.17326

**Published:** 2025

**Authors:** Özlem Özmen, Melda Şahi̇n, Şenay Topsakal, Şerife Taşan, Uğur Şahi̇n

**Affiliations:** 1 Department of Pathology, Faculty of Veterinary Medicine, Burdur Mehmet Akif Ersoy University, Burdur, Türkiye; 2 Department of Endocrinology and Metabolism,University, Pamukkale Faculty of Medicine, Denizli, Türkiye; 3 Genetic Research Unit, Innovative Technologies Application and Research Center, Süleyman Demirel University, Isparta, Türkiye

**Keywords:** Apoptosis, Cytokines, Diabetes mellitus, Inflammation, Lycopene, Signal transduction

## Abstract

**Objective(s):**

Emerging evidence suggests that the JAK/STAT/SOCS signaling pathway is crucial for maintaining homeostasis, and its dysregulation contributes to diabetes development. This study aimed to evaluate the roles of SOCS-1 and SOCS-3 in the pancreas, liver, and kidney and to explore the involvement of the JAK/STAT pathway in the molecular mechanisms underlying their effects on inflammation and apoptosis, as well as organ injury in a diabetes mellitus (DM) model. Additionally, we sought to elucidate the role of the JAK/STAT/SOCS pathway in mediating the effects of lycopene (LYC).

**Materials and Methods:**

Forty Sprague-Dawley rats were divided into control, DM, LYC, and LYC+DM groups. Diabetes was induced in the DM groups using streptozotocin. LYC was administered to the LYC and LYC+DM groups for 30 days. After the study, pancreas, liver, and kidney tissues were analyzed using histopathological, immunohistochemical, and PCR methods.

**Results:**

Significant vacuolization and degenerative changes were observed in the DM group’s pancreatic islet cells. Kidney and liver tissues showed hyperemia, hemorrhage, and degenerative changes. Immunohistochemical analysis revealed increased expression of Cas-3, TNF-α, IFN-α, and IL-6, while IL-10 was significantly reduced in the DM group. PCR analysis showed elevated levels of TNF-α and Cas-3, with decreased SOCS-1 and SOCS-3 expression in the DM group.

**Conclusion:**

This study highlights the therapeutic potential of targeting the JAK/STAT/SOCS pathway with lycopene, demonstrating its promise in mitigating diabetes and related complications.

## Introduction

Diabetes mellitus (DM) is a chronic metabolic disease characterized by hyperglycemia, which can lead to various complications, including those affecting the cardiovascular system, liver, nervous system, and kidneys (1, 2). Hyperglycemia, oxidative stress induced by hyperglycemia, and inflammation are closely interrelated and contribute to the development and progression of diabetic complications (3). The abnormal increase in free radicals and the concurrent reduction in anti-oxidant defense mechanisms caused by DM can lead to cellular organelle damage, increased lipid peroxidation, biomolecular alterations, and the development of insulin resistance (4). Oxidative stress activates pro-inflammatory cytokines and subsequent inflammation, which promotes the production of reactive oxygen species (ROS) (3, 5). ROS are involved in the onset of inflammation in pancreatic islets, and pancreatic cell death inevitably results in loss of insulin secretion by β-cells, which, in turn, exacerbates insulin resistance (6, 7). 

Janus kinase (JAK)/signal transducers and activators of the transcription (STAT) pathway are involved in both signal transduction and gene transcription. It can mediate inflammation, cell proliferation, and the transmission of fibrotic signals, with the JAK/STAT-dependent signaling pathway contributing to the pathogenesis of DM (8, 9). Many of the key pathogenic processes of diabetes are closely associated with JAK/STAT-dependent signaling, including defects in insulin secretion by pancreatic β-cells, impaired insulin sensitivity and utilization by peripheral tissues, and the development of the inflammatory response that contributes to disease progression (10-12). Suppressors of cytokine signaling (SOCS) proteins, initially identified as negative regulators of the cytokine-activated JAK/STAT signaling pathway, play a crucial role in developing insulin resistance and DM (13, 14). SOCS-1 and SOCS-3, members of the SOCS family, regulate the magnitude and duration of JAK/STAT signaling (13). SOCS-1 and SOCS-3 genes are constitutively expressed in pancreatic islets, with their expression increasing following exposure to pro-inflammatory cytokines and during autoimmune inflammatory processes (15).

Various plant metabolites have been utilized in different antidiabetic products as an alternative strategy for treating DM (16-19). Lycopene (LYC) stands out among these natural compounds due to its broad biological activity (20-22). LYC is a carotenoid with potent anti-oxidant, anti-apoptotic, and anti-inflammatory properties, and it also modulates essential metabolic processes in the body (23). LYC has a beneficial effect on pancreatic β-cells and has been reported to reduce vacuolization of the islets of Langerhans and the loss of insulin-secreting cells, leading to decreased blood glucose levels in diabetic rats (24, 25). 

Increasing evidence underscores the importance of understanding the mechanisms by which the JAK/STAT pathway is negatively regulated in the development and progression of diabetes and its associated complications (26). Additionally, SOCS proteins may play a role in the development of diabetes and diabetic complications (2). However, there is currently no information available on the JAK/STAT/SOCS signaling pathway and the effects of LYC on this pathway. The present study aimed to investigate the function of SOCS-1 and SOCS-3 in the pancreas, liver, and kidney and to explore the involvement of the JAK/STAT signaling pathway in the molecular mechanisms underlying the effects of SOCS-1 and SOCS-3 in inflammation and apoptosis, as well as in pancreas, liver, and kidney injury. Additionally, we aimed to elucidate the role of the JAK/STAT/SOCS pathway in mediating the effects of LYC.

## Materials and Methods

### Animals and ethical approval

All animal experiments were conducted following the guidelines for animal research as specified by the Animal Research: Reporting *in vivo* Experiments (ARRIVE 2.0) guidelines and were approved by the Local Committee on Animal Research of Burdur Mehmet Akif Ersoy, Türkiye (MAKU-HADYEK-17.01.2024/1251). 

This study utilized forty Sprague-Dawley rats weighing between 125 and 150 grams. Before starting the experiment, a veterinarian performed health assessments on the rats to ensure their well-being. The rats were provided with a standard commercial diet (Korkuteli Yem, Antalya, Türkiye) and housed at 21 and 22 °C with a humidity level of 60% ± 5% under a 12:12-hour light/dark cycle.

### Experimental procedure

In this study, an experimental diabetes model was created using streptozotocin (STZ). The dosages for LYC and STZ were based on our previous study. After the adaptation period, the rats were randomly divided into four groups, each consisting of ten rats. The groups were as follows;

Control group (n:10): This group of rats received no treatment and was given a standard commercial diet. They were administered an equal volume of citrate buffer.

LYC group (n:10): The rats in this group were administered orally LYC (Sigma Chemical Co) at a dose of 10 mg/kg/day (25, 27) for 30 days.

STZ group (n:10): Rats in this group were administered a single dose of 50 mg/kg STZ (Sigma Chemical Co) dissolved in 0.1 M cold citrate buffer (pH = 4.5) via intraperitoneal injection (IP). Based on our previous studies, this dose was selected to induce incomplete destruction of pancreatic beta cells, which showed that STZ could produce varying degrees of diabetes, from mild to severe, depending on the dose and administration method (25, 28).

LYC + DM group (n:10): Rats in this group were treated with LYC throughout the study and then administered STZ on the 31st day of the experiment to induce DM.

After the study was completed, all rats were anesthetized with Xylazine HCl (Xylasinbio 2%, Bioveta, Czech Republic) and Ketalar HCl (Ketasol, Richter Pharma AG, Austria) and then decapitated to obtain pancreatic samples. The pancreas, liver, and kidney samples were divided into two equal portions: one portion stored at -80 °C for qPCR analysis, and the other was fixed in 10% buffered formalin for histological and immunohistochemical analyses.

### Histopathological method

The collected tissue samples were processed routinely and embedded in paraffin wax using a fully automated tissue processing equipment (Leica ASP300S, Leica Microsystem, Nussloch, Germany). Sections with a thickness of five microns were cut from the paraffin blocks using a fully automatic rotary microtome (Leica 2155, Leica Microsystem, Nussloch, Germany). The samples were then subjected to an alcohol and xylene series and stained with Harris hematoxylin-eosin (HE) (Tek-Path, İzmir, Türkiye) for five and two minutes, respectively. After staining, the samples were mounted on coverslips and examined under a light microscope.

Microscopic changes were assessed blindly. Scores were assigned based on the number of degenerated cells observed in each pancreas, liver, and kidney section: (0 for no degenerated cells; 1 for 1–3 degenerated cells; 2 for 4–7 degenerated cells; and 3 for more than eight degenerated cells. Histopathological changes in each rat were assessed in ten different areas (29). Two experienced pathologists examined each rat’s pancreatic, liver, and kidney samples. Ten Langerhans islets were randomly selected for analysis, and 100 cells were counted using ImageJ 1.48 software (National Institutes of Health, Bethesda, MD, USA). The software’s counter function recorded mouse clicks on cells marked with colorful dots. The final score for each animal was calculated by averaging the two counts.

### Immunohistochemical examination

Five serial sections were taken from the previously prepared paraffin blocks and placed on poly-L-lysine-coated slides. Immunohistochemical staining was performed to detect the expression of Caspase-3 [Recombinant Anti-Caspase-3 p12 antibody [EPR16888] (ab179517)], TNF-alpha [TNF-α Recombinant antibody [RM1005] (ab307164)], IFN-α [Anti-Interferon alpha 2 antibody (ab193055)], IL-6 [Anti-IL-6 antibody [1.2-2B11-2G10] (ab9324)], and IL-10 [Anti-IL-10 antibody (ab217941)] (Abcam, Cambridge, UK) using the streptavidin–biotin technique according to the manufacturer’s instructions. All primary antibodies were used at a 1/100 dilution with antibody dilution solution (ThermoFisher Scientific, MA, USA), and sections were incubated with these antibodies for 60 min. Immunohistochemistry was then performed using a biotinylated secondary antibody and streptavidin-alkaline phosphatase conjugate. Diaminobenzidine (DAB) was used as the chromogen for HRP/DAB detection specific to rabbits, with the secondary antibody being from the IHC Detection Kit - Micro-polymer (ab236469) and DAB Substrate Kit (ab64238), (Abcam, Cambridge, UK). DAB was applied to the sections for three to five minutes. Antigen dilution solution was used instead of the primary antibody for negative controls. Two expert pathologists independently and blindly conducted all evaluations. For each antibody, sections were examined separately for immunohistochemical analysis, and the percentage of positive cells on each slide was assessed.

A comparison was made between the counts of positive cells in the pancreatic tissue of the control group, the total number of positive cells per high-power field, and the number of positive cells for each marker per islet. At least five high-power fields (X40) were selected for each islet. Immunohistochemical investigations were performed using ImageJ 1.48 (National Institutes of Health, Bethesda, MD, USA), and statistical analysis was based on the output from the image analyzer. Microphotography was performed using the Database Manual Cell Sens Life Science Imaging Software System (Olympus Co., Tokyo, Japan).

### The reverse transcription-polymerase chain reaction (RT-qPCR)

RNA was extracted from homogenized pancreas, liver, and kidney tissues using the RNA Isolation Kit-Tissue and Cell Culture (Hibrigen, Türkiye) and the manufacturer’s instructions. MySPEC microvolume spectrophotometer (VWR, USA) instrument was used to measure the amount and purity of the collected RNAs. For cDNA synthesis, 1 µg of total RNA was reverse transcribed into cDNA using iScript Reverse Transcription Super Mix according to the manufacturer’s instructions. All cDNA samples were stored at −20 °C until RT-qPCR analysis. The cDNA synthesis step was performed on the C1000 thermal cycler (BioRad, CA, USA).

RT-qPCR amplification was performed with Sso Advanced Universal SYBR Green Supermix (BioRad, CA, USA), and a fluorescent signal was detected on a CFX 96 instrument (BioRad, CA, USA). Four reference genes that are commonly used—TNF-α, Caspase-3 (Cas-3), SOCS-1, and SOCS-3—were selected for this study. Housekeeping gene GAPDH was used for the study. Specific mRNA sequences were found using the Primer-BLAST tool and the National Center for Biotechnology Information (NCBI) website, and potential primer sequences were tested. In Table 1, the primers’ sequences were given. cDNA samples were run in triplicate for each parameter. GAPDH expression was used for data normalization. The following qPCR conditions were used: pre-denaturation at 95 °C for 10 min followed by 35 cycles of 10 sec at 95 °C and 30 sec at 59 °C. 100 ng of cDNA was used as a template in a total volume of 25 µl. Relative mRNA levels were calculated by applying the 2^−ΔΔC^t formula to the normalize the result (30). Melting curve analysis was evaluated to determine the specificity of the amplification. The results are shown in the graph as fold change.

### Statistical analysis

We conducted a power analysis using G* Power Version 3.1.9.7 to determine the minimum sample size required before the study. The parameters used were effect size = 0.8, alpha = 0.08, anticipated power (1-beta) = 0.95, and the number of groups set to 7. Ten animals were assigned to each group to avoid potential disruptions in statistical analyses due to animal deaths. Presentation formats for variables included means and standard deviations, medians, minimum-to-maximum values, frequencies, percentages, and medians.

We used the Statistical Package for Social Sciences (SPSS) 22.00 (SPSS Inc., Chicago, IL, USA) for statistical analysis. The data were initially assessed for normality of distribution using the Shapiro-Wilk test. Since the data showed a normal distribution (*P*>0.05), comparisons between groups were performed using a one-way analysis of variance (ANOVA). Data with a normal distribution were presented as mean and standard deviations. Immunohistochemical analysis was performed on the percentage of immunopositive cells. Group differences were assessed using a one-way ANOVA followed by the Duncan post hoc test, with statistical significance at *P*<0.05.

Genetic data were analyzed using SPSS 20.0 (IBM Corporation, Chicago, USA). The data were examined across groups using one-way ANOVA, followed by the LSD multiple comparison test. Results are reported as mean ± standard deviations (SD). The threshold for statistical significance was considered at *P*<0.05.

## Results

### Histopathological and immunohistochemical findings in pancreas

**Figure 1:**
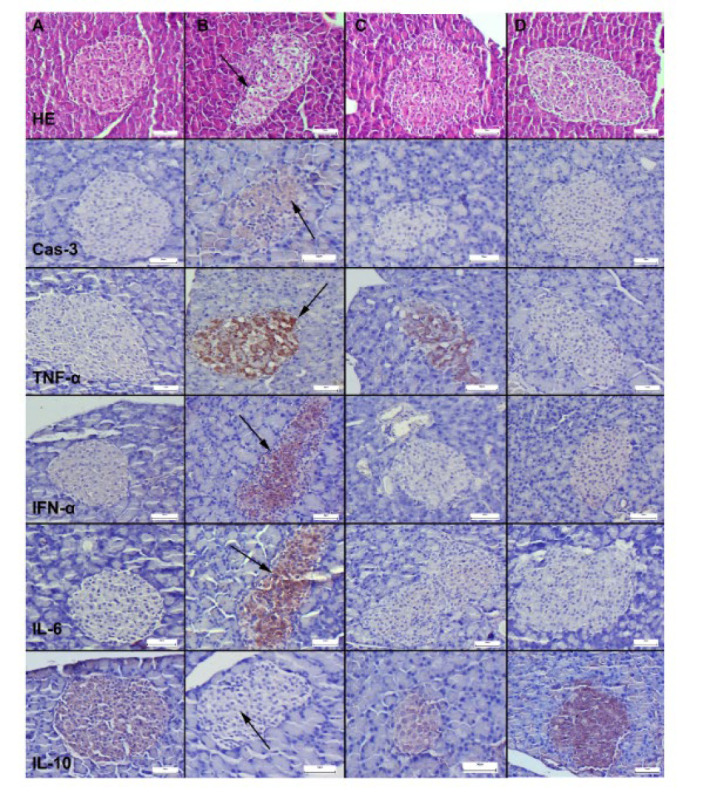
Microscopic images of the Langerhans islets in the pancreas across different groups in rats (first row)

Histopathological examination revealed normal pancreatic histology in the control and LYC groups. However, in the DM group, significant vacuolization and degenerative changes were observed in the cells of the pancreatic islets, with these changes being concentrated in the central parts of the islets. Notably, the histopathological findings significantly improved in the LYC+DM group (Figure 1).

Immunohistochemical examinations showed a significant increase in the expression of Cas-3, TNF-α, IFN-α, and IL-6 in the pancreatic islets of the DM group. Conversely, the expression of IL-10 decreased markedly in this group. LYC treatment effectively restored all markers’ expressions to normal levels (Figure 1). 

**Figure 2:**
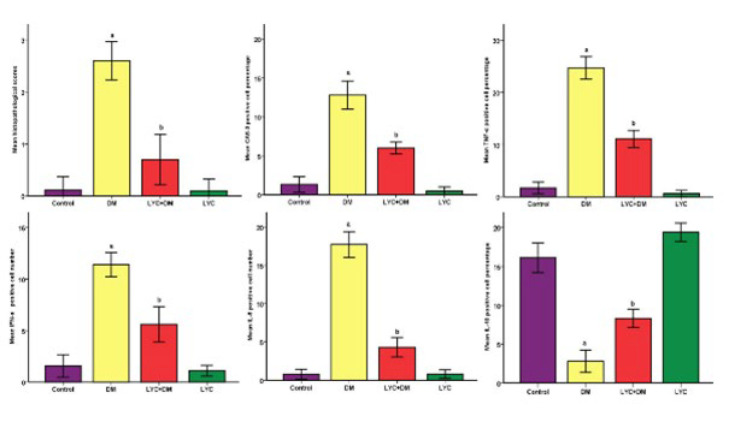
Statistical analysis of histopathological scores and the percentage of immunohistochemically positive cells in the pancreas in rats

The statistical analysis results for histopathological scores and percentage of immunopositive cells are shown in Figure 2.

### Histopathological and immunohistochemical findings in kidney and liver

**Figure 3:**
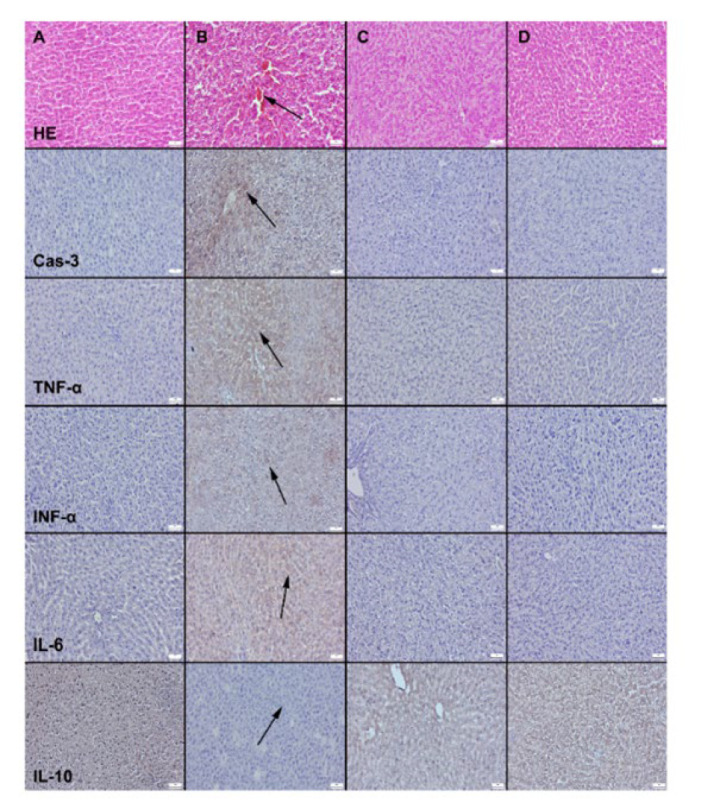
Representative microscopic appearance of the livers across different groups in rats(first row)

**Figure 4:**
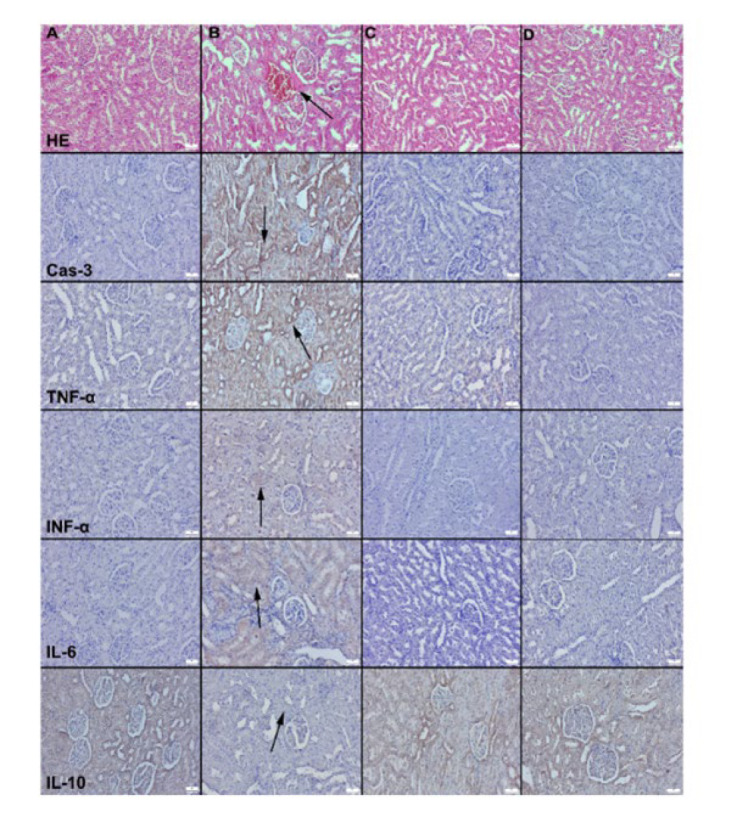
Microscopic images of the kidneys across different groups in rats (first row)

In the kidney tissues of the control and LYC groups, normal glomerular structure and interstitium were observed, with no inflammatory cell infiltration in the interstitium. In contrast, the kidney tissues of the DM group exhibited hyperemia, hemorrhage, and degenerative changes (Figure 4). LYC treatment led to a reduction in these pathological findings in the LYC+DM group. In the liver tissue, the histology of the liver in the control and LYC groups showed a normal, clear, and typical liver lobular architecture along with normal hepatocyte structure. Conversely, the DM group displayed increased hyperemia, hemorrhages, and degenerative change. However, these pathological findings were significantly reduced, evident in the LYC+DM group (Figures 3 and 4).

Immunohistochemical examinations revealed a significant increase in the expression of Cas-3, TNF-α, IFN-α, and IL-6 in the liver and kidney of the DM group. Conversely, the expression of IL-10 decreased markedly in this group. LYC treatment effectively restored all markers’ expressions to normal levels (Figures 3 and 4).

### mRNA expression analysis findings

**Figure 5:**
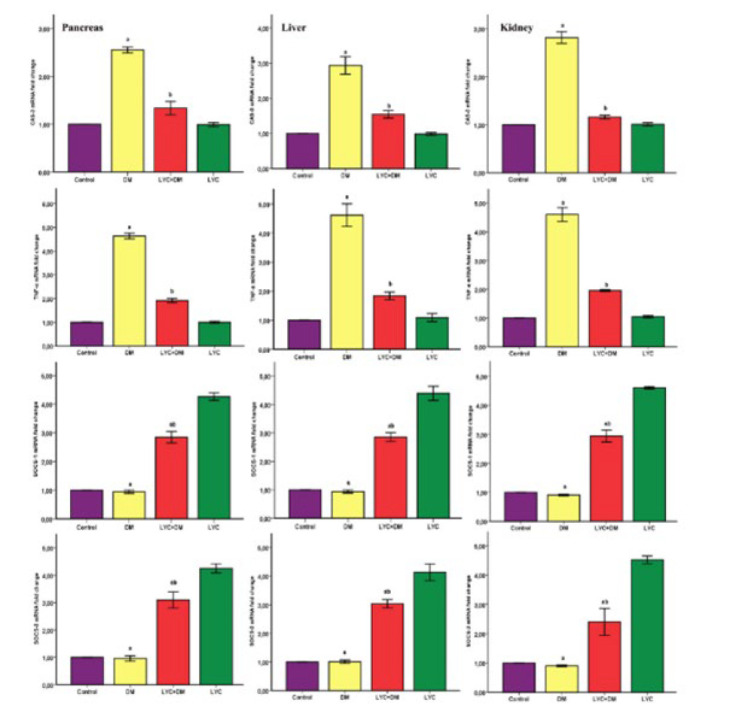
Effects of lycopene on the mRNA expression levels of Cas-3, TNF-α, SOCS-1, and SOCS-3 in tissues from the pancreas, liver, and kidney of rats

The expression levels of genes associated with inflammation and apoptosis were compared between groups. In the DM group, there was an increase in the expression levels of the TNF-α and Cas-3 genes compared to the control group and a significant decrease in the expression levels of SOCS-1 and SOCS-3 genes (*P*<0.05). In the LYC + DM group treated with LYC, the expression levels of these genes were significantly altered to those in the control group (*P*<0.05) (Figure 5). Based on these results, it was determined that LYC reduced the inflammation and apoptosis induced by DM in the pancreas, liver, and kidney tissues.

## Discussion

The JAK/STAT/SOCS signaling pathway is crucial in regulating various cellular processes, including immune function and metabolism. Diabetes is also related to immune function, and it is believed that the JAK/STAT/SOCS signaling pathway may significantly impact the development of diabetes due to its influence on insulin signaling and inflammation. Dysregulation of the JAK/STAT pathway is thought to contribute to insulin resistance, a hallmark of type 2 diabetes (31). Additionally, SOCS proteins, which act as negative regulators of this pathway, are often upregulated in diabetes, exacerbating insulin resistance and inflammatory responses (32). However, these mechanisms are not fully understood. A comprehensive understanding of this pathway is crucial for developing effective therapeutic strategies for diabetes management. Further research is needed to prevent, treat, or alleviate the symptoms of diabetes to improve patients’ quality of life. 

LYC, a naturally occurring carotenoid found in tomatoes and other red fruits, has demonstrated significant potential in ameliorating diabetes-induced damage in pancreatic, hepatic, and renal tissues (25, 33-35). The JAK/STAT/SOCS signaling pathway is critical in the inflammatory response and cellular stress associated with diabetes. This study was designed with the hypothesis that LYC can inhibit the JAK/STAT/SOCS signaling pathway, thereby reducing inflammation and insulin resistance. Our study results indicate that LYC inhibits this pathway in the pancreas, liver, and kidneys, significantly mitigating the progression of diabetic complications. Therefore, LYC represents a promising therapeutic or prophylactic agent for managing DM and its associated organ damage.

The relationship between DM and immunity is well established (36, 37). Cytokines are proteins produced by various cell types, either constitutively or upon activation, that act as molecular mediators of innate and adaptive immunity. They typically function as intermediaries within and between these subsystems (38). Cytokine families include chemokines, which induce chemotaxis; interleukins, which are involved in pathogen elimination; and proliferation of immune cells; interferons, which are primarily involved in pathogen elimination; and the tumor necrosis factor (TNF-α) family, which regulates immune cell functions (39-41). In diabetic rats, cytokines such as IFN-γ, IL1, IL-6, IL-8, and TNF-α have been shown to be induced (42-44). In diabetes, the hyperglycemic microenvironment, characterized by dysregulation cytokine secretion and expression, leads to low-grade cellular activation and inflammation. This can persist and contribute to the onset and progression of diabetic complications, including diabetic liver disease and nephropathy (45). Growing evidence suggests that type 1 IFN (IFN-α) plays a significant role in this process. The production of the potent immunomodulator IFN-α is usually typically initiated early in the innate immune response, making it a crucial factor in the downstream redirection of both innate and adaptive immunity (46, 47). 

Numerous studies have shown that LYC modulates inflammation in diabetic rats by inhibiting cytokines such as IFN-γ, IL1, IL6, IL8, and TNF-α (24, 48-50). Our study results indicate that in diabetic rats, LYC reduces the expression of Cas-3, IFN-α, IL-6, and TNF-α, crucial cytokines in the pathogenesis of diabetes. This finding confirms that LYC can reduce the inflammatory response by suppressing cytokine secretion in diabetic rats, thereby alleviating damage to the pancreas, liver, and kidneys.

The production of pro-inflammatory cytokines at high levels is associated with inflammation, which is mediated through the JAK/STAT signaling pathways (51). Activation of the JAK/STAT pathway also induces the expression of SOCS proteins, which act as internal suppressors of the JAK/STAT pathway. Therefore, the JAK/STAT pathway and SOCSs can be considered a negative feedback system (13). Overexpression of SOCS inhibits the insulin signaling system by blocking the binding of insulin receptor substrates (IR) to the insulin receptor (IR), leading to insulin resistance (32, 51). Our study found that SOCS-1 and SOCS-3, two members of the SOCS family, were overexpressed in the LYC+DM group. This may contribute to reduction in the pathological changes associated with pancreatic, hepatic, and renal injury. Additionally, our study demonstrated that lycopene effectively treats DM due to its anti-oxidant, anti-apoptotic, antifibrotic, and anti-inflammatory mechanisms.

Hyperglycemia is a hallmark of the complex, multi-organ metabolic disease known as DM. Recent research indicates that the highly conserved and potent JAK/STAT signaling system is crucial for maintaining homeostasis and plays a role in developing DM and obesity when dysregulated (52). This study examines the role of JAK/STAT activation and SOCS inhibition in the pancreas, liver, and kidney and how these factors impact the progression of the disease. We also discuss the therapeutic implications of targeting the JAK/STAT/SOCS signaling pathway in treating DM and obesity.

 A deficiency in pancreatic β-cell is a common characteristic of both type 1 diabetes (T1D) and type 2 diabetes (T2D). While the cause of β-cell dysfunction and death differ between the two forms of diabetes, JAK/STAT signaling involves both processes. In T1D, autoimmune mechanisms lead to the destruction of insulin-producing β-cells. In T2D, β-cells fail to secrete sufficient insulin, leading to insulin resistance in peripheral tissues. Therefore, the loss of functioning β-cells is a key factor in developing DM and hyperglycemia (52). In this study, we evaluated and confirmed the effect of STZ-induced DM and the protective effects of LYC through histopathological, immunohistochemical, and PCR analysis.

Immune cells in the islets of T1D release pro-inflammatory cytokines that affect β-cell gene transcription, leading to β-cell dysfunction and death (53). β-cell apoptosis is induced when tumor necrosis factor α or IL-1β, along with IFN-γ, is applied to human and animal islets and insulinoma cells (54, 55). In this study, β cell degeneration and changes in pancreatic islets were clearly observed at the cellular level following STZ administration. Additionally, the islet-protective effects of LYC were distinctly demonstrated. The positive effects of LYC on DM, observed in our previous similar study, are now supported at the molecular level, reinforcing the data in this area.

It is known that DNA binding elements and upstream activators interact with STAT1, STAT3, and STAT5. The importance of STAT protein production and activation for β-cell biology is significant. Additionally, IFN signaling also influences this production (56). Maintaining a normal β-cell population and function requires regulating pro- and anti-inflammatory responses and STAT activation. In T1D patients, immune cells also contribute to a highly oxidative microenvironment in the pancreas (57). The findings of this study are consistent with previous research.

A primary limitation of this study is that only a single dose of LYC was used. Future studies could explore different doses and durations to provide a more comprehensive understanding of LYC’s effects.

**Table 1:** Specific primers of this study in rats

Primers	Specific primer sequence
SOCS-1	F: TAACCCGGTACTCCGTGACT
	R:CTCCCACGTGGTTCCAGAAA
SOCS-3	F: CTGGACCCATTCGGGAGTTC
	R:AACTGGGAGCTACCGACCAT
TNF-α	F: TCGTCTACTCCTCAGAGCCC
	R: ACTTCAGCGTCTCGTGTGTT
Cas-3	F:GAGCTTGGAACGCGAAGAAA
	R: TTGCGAGCTGACATTCCAGT
GAPDH (HouseKeeping)	F:CAGCCGCATCTTCTTGTGC
	R:TACTCAGCACCAGCATCACC

GAPDH: Glyceraldehyde-3-phosphate dehydrogenase; SOCS-1: Suppressor of cytokine signaling 1; SOCS-3: Suppressor of cytokine signaling 3; TNF-α: Tumor necrosis factor-alpha

(A) Normal tissue histology in the control group, (B) Significant vacuolization in the cells (arrows) in the DM group, (C) Normal islet appearance in the LYC+DM group, (D) Normal tissue histoarchitecture in the LYC group, HE. Immunohistochemical findings for Cas-3, TNF-α, IFN-α, IL-6, and IL-10 in the pancreas across the groups, with arrows indicating cell changes. Streptavidin biotin peroxidase method, Scale bars=50 μm

(1A) Normal liver tissue histology in the control group. (1B) Severe hyperemia, hemorrhages, and degenerative change (arrows) in the DM group. (1C) Normal liver histology in the LYC+DM group. (1D) Normal liver tissue appearance in LYC group, HE. Immunohistochemical findings for Cas-3, TNF-α, IFN-α, IL-6, and IL-10 in liver tissue across the groups. Arrows indicate cell changes, Streptavidin biotin peroxidase method, Scale bars=50 μm.

(1A) Normal kidney tissue histology in the control group. (1B) Severe hyperemia, hemorrhages, and degenerative change (arrows) in the DM group. (1C) Normal kidney histology in the LYC+DM group. (1D) Normal kidney tissue appearance in LYC group, HE. Immunohistochemical expressions of Cas-3, TNF-α, IFN-α, IL-6, and IL-10 in the kidneys across the groups. Arrows indicate changes in the cells, Streptavidin biotin peroxidase method, Bar=50 μm.

Letters a and b denote statistically significant differences between control and DM groups, respectively, at *P*<0.05.

## Conclusion

LYC shows significant potential in ameliorating diabetes-induced damage to the pancreas, liver, and kidneys by modulating the JAK/STAT/SOCS signaling pathway. By inhibiting this pathway, LYC reduces cellular damage and mitigates the progression of diabetic complications. These findings highlight LYC’s therapeutic or prophylactic potential in managing diabetes and its associated organ damage, positioning it as a beneficial dietary component for improving overall metabolic health. Further research and clinical studies are needed to fully elucidate the mechanisms and optimize the therapeutic applications of lycopene in diabetes care.

## Declaration

We have not used any AI tools or technologies to prepare this manuscript.
